# A Signal-to-Noise Crossover Dose as the Point of Departure for Health Risk Assessment

**DOI:** 10.1289/ehp.1003327

**Published:** 2011-08-03

**Authors:** Salomon Sand, Christopher J. Portier, Daniel Krewski

**Affiliations:** 1 Risk Benefit Assessment Department, National Food Administration, Uppsala, Sweden; 2 McLaughlin Centre for Population Health Risk Assessment, University of Ottawa, Ottawa, Ontario, Canada; 3 National Institute of Environmental Health Sciences, National Institutes of Health, Department of Health and Human Services, Research Triangle Park, North Carolina, USA; 4 Risk Sciences International, Ottawa, Ontario, Canada

**Keywords:** benchmark dose, cancer bioassay, human exposure guideline, low-dose extrapolation, point of departure, reference dose, signal-to-noise crossover dose, uncertainty factor

## Abstract

Background: The U.S. National Toxicology Program (NTP) cancer bioassay database provides an opportunity to compare both existing and new approaches to determining points of departure (PoDs) for establishing reference doses (RfDs).

Objectives: The aims of this study were *a*) to investigate the risk associated with the traditional PoD used in human health risk assessment [the no observed adverse effect level (NOAEL)]; *b*) to present a new approach based on the signal-to-noise crossover dose (SNCD); and *c*) to compare the SNCD and SNCD-based RfD with PoDs and RfDs based on the NOAEL and benchmark dose (BMD) approaches.

Methods: The complete NTP database was used as the basis for these analyses, which were performed using the Hill model. We determined NOAELs and estimated corresponding extra risks. Lower 95% confidence bounds on the BMD (BMDLs) corresponding to extra risks of 1%, 5%, and 10% (BMDL_01_, BMDL_05_, and BMDL_10_, respectively) were also estimated. We introduce the SNCD as a new PoD, defined as the dose where the additional risk is equal to the “background noise” (the difference between the upper and lower bounds of the two-sided 90% confidence interval on absolute risk) or a specified fraction thereof.

Results: The median risk at the NOAEL was approximately 10%, and the default uncertainty factor (UF = 100) was considered most applicable to the BMDL_10_. Therefore, we chose a target risk of 1/1,000 (0.1/100) to derive an SNCD-based RfD by linear extrapolation. At the median, this approach provided the same RfD as the BMDL_10_ divided by the default UF.

Conclusions: Under a standard BMD approach, the BMDL_10_ is considered to be the most appropriate PoD. The SNCD approach, which is based on the lowest dose at which the signal can be reliably detected, warrants further development as a PoD for human health risk assessment.

Since the introduction of the benchmark dose (BMD) by Crump (1984), there has been considerable discussion about the merits of this measure relative to the traditional no observed adverse effect level (NOAEL), originally introduced by Lehman and Fitzhugh (1954) as a point of departure (PoD) for establishing human exposure guidelines, expressed in the form of a reference dose (RfD) (Barnes and Dourson 1988).

In its simplest form, the RfD is determined by the formula RfD = PoD/UF, where the uncertainty factor (UF) denotes the combined effect of inter- and intraspecies differences, as well as other uncertainties associated with the available data (Dourson et al. 1996). Essentially the same approach is used to derive other exposure guidelines, such as the tolerable daily intake or acceptable daily intake, but here we use the term “RfD” to represent a human exposure guideline, regardless of the type of test agent or critical end point considered.

In practice, a lower confidence limit on the BMD (the BMDL) is used as the PoD when the BMD approach is invoked. The introduction of the BMD for noncancer end points has increased comparability between approaches to estimate exposure guidelines for noncancer and cancer agents. Currently, a single approach is generally recommended to derive the PoD, but differences exist in terms of how to proceed after the derivation of the PoD, depending on whether the agent is nongenotoxic or a genotoxic carcinogen [European Food Safety Authority (EFSA) 2005, 2009a; U.S. Environmental Protection Agency (EPA) 2005]. Several authors have specifically suggested that the BMDL may serve as a PoD for carcinogens and noncarcinogens alike, leading to an integrated approach to cancer and noncancer risk assessment (Gaylor et al. 1999; National Research Council 2009).

Potential advantages of the BMDL relative to the NOAEL include the use of all available data in its determination and avoidance of the restriction that it be equal to one of the experimental doses. Although the BMD concept is best developed for quantal response data (i.e., presence or absence of a given response), methods have also been developed for continuous response data (Gaylor et al. 1998; Murrell et al. 1998; Sand et al. 2006; Slob 2002; Slob and Pieters 1998), and default approaches for applying the BMD concept for either type of response data have been suggested (EFSA 2009a; U.S. EPA 2000). [For more information on approaches for continuous and quantal data and a detailed comparison of the BMD vs. NOAEL, see EFSA (2009a) and Sand et al. (2008).]

In the past, the BMD method was employed primarily by regulatory authorities in the United States, such as the U.S. EPA. However, the EFSA (2009a) recently released a scientific opinion that recommends the BMD approach for setting human exposure guidelines, which has increased use of the BMD in risk assessments performed by the EFSA (2009b, 2009c, 2010).

A crucial aspect of the BMD method is the specification of the benchmark response (BMR) level associated with the BMD. For quantal data, the BMD with BMDL was originally presented as the dose causing an excess risk in the range of 1–10% (Crump 1984; Kimmel and Gaylor 1988). In its opinion on the BMD, the EFSA (2009a) argued that the BMR should ideally represent an effect size that is negligible or nonadverse, with the practical constraint that the BMR cannot be set at levels that are so low (or high) that estimation of the BMD requires extrapolation beyond the range of the experimental data, in which case the result may be highly model dependent. Therefore, for quantal data representing severe lesions, the desirable or acceptable risk level is often much lower than what could be employed as a BMR, given the practical constraints of the data.

The issue of what BMR may be appropriate as a starting point has been addressed to a certain extent by studies that have compared actual NOAEL and BMDL values across several data sets. Results suggest that the average risk at the NOAEL may be in the range of 5–10% (Allen et al. 1994b; Fowles et al. 1999) or even > 10% (Allen et al. 1994a), and use of a BMR in this range has been suggested. The U.S. EPA has used an extra risk of 10% as a default (with the exception of developmental toxicity data, where an extra risk of 5% has been applied), and the EFSA also recommends a default BMR (extra risk) of 10% for quantal data, with modification based on statistical or toxicological considerations as needed (EFSA 2009a).

The derivation of a PoD under a dose–response modeling approach is closely related to the selection of the BMR. We investigated the question of which BMR is most closely associated with the traditional PoD—the NOAEL—in detail using an analysis that provides a benchmark against which new approaches can be compared. Motivated in part by practical constraints on the lower end of the range of possible BMR values, we present a new strategy for derivation of the PoD based on the concept of a signal-to-noise crossover dose (SNCD). The SNCD is defined as the dose at which the additional risk equals the “background noise” or some fraction (e.g., 0.67) of the noise, where “background noise” is defined as the difference between the upper and lower bounds of the two-sided 90% confidence interval (CI) on absolute risk. The SNCD thus provides an estimate of the lowest dose that can be derived as a PoD for risk assessment without low-dose extrapolation. The SNCD is compared with typical target PoDs, specifically, the BMDLs corresponding to extra risks of 1%, 5%, and 10% (BMDL_01_, BMDL_05_, and BMDL_10_, respectively) and the NOAEL. In addition, we describe an approach for deriving an RfD using the SNCD as the PoD and compare the resulting RfD to RfDs derived from the NOAEL and BMDL. We used the complete U.S. National Toxicology Program (NTP) database on 2-year carcinogen bioassays in rats and mice (NTP 2009) as the basis for these analyses.

## Materials and Methods

*Databases.* We used data from the U.S. NTP 2-year rodent toxicology and carcinogenesis studies to evaluate the different PoDs and RfDs. Since 1981 the NTP has directed a rodent carcinogenicity bioassay program begun by the National Cancer Institute (NCI). Together the NCI and NTP have conducted > 500 cancer studies. Most of these studies follow a relatively common protocol, with male and female rats and mice tested at a control and three dose levels for each test agent (Bucher and Portier 2004; Chhabra et al. 1990).

Data were abstracted on 13–14 January 2009 from the Toxicology Database Management System (TDMS) and Carcinogenesis Bioassay Data System (NTP 2009). Only studies marked as “chronic” in the TDMS were included (204 rat studies and 192 mouse studies). Individual animals were excluded from the data set if they were designated as “interim/scheduled sacrifice,” “missing,” “wrong sex,” and “other,” and dose groups that were marked as sentinel (SEN) or restricted diet (RTD) were excluded. As in the regular NTP technical reports summarizing the study results, all dose groups with the same concentration values (other than stop-dose groups) were automatically merged. Stop-dose groups were excluded from trend statistics but were included in pairwise statistics.

*Data selection.* The data generated as described above were considered for inclusion in the present analysis if they satisfied the following three criteria: *a*) The data for the end point demonstrated a significantly increasing dose–response trend according to the NTP poly3 *p*-value for trend (*p* ≤ 0.05); *b*) the NTP determined that the end point demonstrated “some” or “clear” evidence of carcinogenicity; and *c*) the data for the end point demonstrated a NOAEL according to the NTP poly3 *p*-value (*p* > 0.05). (Within a given data set, the highest nonsignificant dose was regarded as the NOAEL for that data set.) These criteria resulted in the initial selection of 1,383 individual data sets. We excluded any stop doses and excluded data sets describing combinations of morphology codes (e.g., “adenoma and carcinoma”) if the combined response was ascribed only to the observed response associated with a specific morphology code (e.g., “adenoma” response but zero “carcinoma” response) already accounted for in the data selection. These refinements yielded 1,128 data sets spanning 140 technical reports. The number of individual data sets in the 140 reports ranged from 1 to 53, with a median number of 6. Results presented in the present study are based on the data sets fulfilling the requirements discussed above (1,128 data sets), along with standard requirements for BMD analysis, as described below.

*Model fitting.* The dose–response data were fitted with the Hill model by maximum likelihood; both three- and two-parameter versions of the model were considered. Data sets demonstrating a significant dose–response trend and adequate goodness of fit (both *p* ≤ 0.05, consistent with standard data set requirements for calculating a BMD) were included in the analyses, leaving a total of 786 data sets. For a subset of these data, model dependence was investigated by contrasting Hill model results with estimates from a Weibull model. Technical details regarding the models, the model fitting, the model selection process, and the analysis of model dependence are provided in Supplemental Material, Section 1 (http://dx.doi.org/10.1289/ehp.1003327).

*The risk at the PoD.* Using the profile likelihood method, the upper (one-sided) 95% confidence bound on the extra risk (UER) at a given dose, *d*, will, if used as the BMR in a standard BMD analysis, result in a BMDL that equals dose *d* (i.e., if the BMDL is defined as the lower one-sided 95% confidence bound on the BMD and is estimated under the profile likelihood method). We used the fitted Hill model to obtain a point estimate, as well as an upper 95% confidence bound, on extra risk at the NOAEL for each data set. The upper 95% confidence bound on extra risk (UER) at the NOAEL (UER_NOAEL_) (estimated using the profile likelihood method) was compared with standard BMRs (extra risks) of 1%, 5%, and 10% used in BMD analysis.

A central estimate of the UER_NOAEL_ may be regarded as a reflection of the typical level of risk associated with the traditional PoD used in risk assessment. Extra risk calculations identical to those described for the NOAEL were also performed for the SNCD.

*The signal-to-noise crossover dose.* The SNCD is defined as the dose where the point estimate of additional risk is equal to or, alternatively, 0.67× the (absolute) difference between the upper and lower bound of a two-sided 90% CI on absolute risk at that dose. In the process of estimating the SNCD, a signal-to-noise ratio (SNR) was defined as

**Figure 1:**
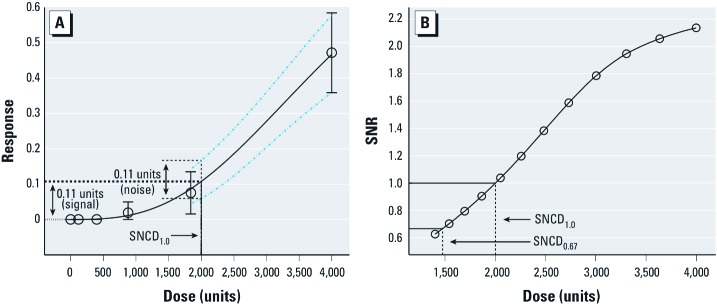
Estimation of the SNCD. (*A*) The Hill model fitted to data on liver cholangiocarcinoma observed in female Harlan Sprague-Dawley rats exposed to 2,3,7,8-tetrachlorodibenzo-*p*-dioxin (solid curve) (NTP 2006). The dotted curves represent the two-sided 90% CI on absolute risk around the fitted model, as estimated by the profile likelihood method; circles represent observed responses (in this example, for six different doses) with 90% CIs. SNCD_1.0_ represents the dose (2,000 units) at which the additional risk (0.11 – 0 = 0.11 units: the signal) is equal to the (absolute) difference between the upper (0.17) and lower (0.06) bound on absolute risk (0.17 – 0.06 = 0.11 units: the noise). The SNCD_1.0_ corresponds to an SNR equal to 1 (0.11/0.11 = 1). (*B*) The relationship between dose and SNR. The SNR at a given dose *d* equals the point estimate of additional risk at *d* divided by the (absolute) difference between the upper 95th and lower 5th confidence bound on absolute risk at *d*. The doses that correspond to SNRs of 1 and 0.67 are solved from the spline function as the SNCD_1.0_ and SNCD_0.67_, respectively.

SNR = [*p*(*d*) – *p*(0)]/(P95 – P05), [1]

where *p*(*d*) – *p*(0) is the point estimate of additional risk at dose *d*, and P95 – P05 is the difference between the upper 95th and lower 5th confidence bound on absolute risk at dose *d*. We defined two SNCDs, the SNCD_1.0_ (corresponding to a critical SNR of 1) and the SNCD_0.67_ (corresponding to an SNR of 0.67). We estimated the SNCD using the profile likelihood method, by evaluating the SNR at progressively lower doses until the critical SNR was reached (the SNR approaches zero when the dose approaches zero), as illustrated conceptually in Figure 1. Technical details, including a description of the algorithm used for deriving the SNCD, are provided in Supplemental Material, Section 2 (http://dx.doi.org/10.1289/ehp.1003327).

If the SNCD was outside of the experimental dose range for a data set (i.e., the SNCD was higher than the highest dose), the data set was not used for SNCD estimation. Therefore, the SNCD_1.0_ was derived for only 439 data sets, and the SNCD_0.67_ was derived for 665 data sets. In contrast, the BMDL_10_ was lower than the highest dose for 694 data sets [the point estimate (BMD_10_) was lower than the highest dose for 546 data sets], and the NOAEL was lower than the highest dose for 596 data sets. Under the model selection approach used [see Supplemental Material, Section 1 (http://dx.doi.org/10.1289/ehp.1003327)], the three-parameter Hill model was selected for 62 data sets, including 4 (6%) for which the SNCD_1.0_ was outside the experimental dose range, and the two-parameter Hill model was selected for 724 data sets, including 343 (47%) with SNCD_1.0_ outside of the experimental range. The SNCD was less likely to be out of the experimental range for data sets modeled using a three-parameter model because these data sets usually had more pronounced dose responses.

In addition to calculating the SNCD, we also calculated a point estimate of the extra risk at the SNCD and an upper (one-sided) 95% confidence bound on extra risk at the SNCD (UER_SNCD_). This was done in a manner identical to that described above. The UER_SNCD_ may be regarded as an objective estimate of the lowest practical BMR that may be used for a given data set. The SNCD_1.0_ and SNCD_0.67_ were compared with the NOAEL and BMDLs, corresponding to extra risks of 1%, 5%, and 10%, calculated for each data set for which these measures could be determined.

*Establishment of human exposure guidelines.* It has been debated whether or not the standard UFs used in risk assessment require modification when a BMDL is used as the PoD, rather than a NOAEL. The default UF of 10 × 10 = 100 allows for a possible 10-fold increased sensitivity in humans compared with experimental animals and for a 10-fold variation in susceptibility within the human population. The size of the UF covering such generally unknown variability is, in a strict sense, independent of the BMR used for deriving the PoD from experimental data, because the variability intended to be accounted for spans the entire dose range.

Within the construct of the BMD, the range of BMRs associated with the NOAEL appears to have been, in some sense, regarded as “acceptable.” The specification of a BMR under a quantitative dose–response modeling approach highlights the issue of whether or not an additional UF representing low-dose extrapolation should be added, in addition to accounting for interspecies differences in sensitivity and interindividual variability within the human population. This concerns the case of more severe lesions for which the “acceptable risk” is lower than the BMR value that can be used given the practical data limitations; the UER_SNCD_ may be regarded as an objective estimate of the lowest practical BMR for a given data set.

The choice of a specific UF to take into account the fact that the PoD is not risk free will be influenced by shape of the dose–response curve below the PoD. If the dose–response curve is linear, this factor will reduce the BMR at the PoD proportionally, but nonlinear approaches may also be envisioned. A fundamental reassessment of the UFs to be used is outside the scope of this article. For purposes of comparison of the NOAEL, BMDL, and SNCD as alternative PoDs, it is assumed that the standard UF of 100 is appropriate for establishing an RfD. For comparison purposes, we considered the standard UF of 100 to be most applicable to a BMDL corresponding to the median UER_NOAEL_ (BMR_m_). Using an extra risk of BMR_m_ would provide a similar level of risk protection as the traditional approach that uses a NOAEL as the PoD. Thus, in the present application of the BMD approach, the BMDL corresponding to the BMR_m_ is regarded as an appropriate default PoD to which the current UFs can be applied for purposes of establishing a human exposure guideline.

Application of the default UF of 100 to the dose corresponding to the BMR_m_ translates to a target (extra) risk of BMR_m_/100 in animals if the dose–response curve is linear below the PoD. Using the UER_SNCD_ as the starting point, the RfD corresponding to a target risk of BMR_m_/100 under the assumption of linearity is calculated as

RfD_SNCD_ = (BMR_m_/100) × (SNCD/UER_SNCD_). [2]

This definition implies that the UF applied to the SNCD will depend on the extra risk associated with the SNCD and is thus specific to the data set being analyzed. We used a target risk of BMR_m_/100 and a linear model to determine SNCD-based RfDs as a basis for comparisons with BMDL_10_- and NOAEL-based RfDs derived using a standard UF approach. However, alternative target risks and low-dose extrapolation models also may be considered under the SNCD approach.

## Results

**Figure 2:**
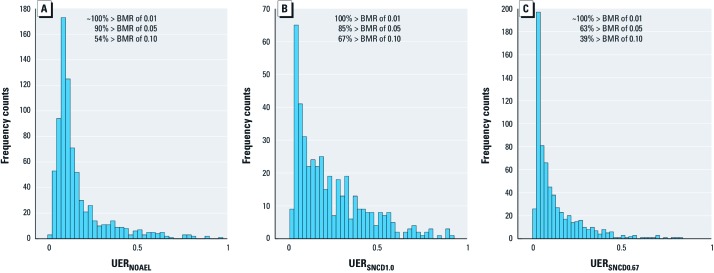
The UER at the NOAEL (*A*), SNCD_1.0_ (*B*), and SNCD_0.67_ (*C*). The percentages of data sets exceeding standard BMR (extra risk) levels of 0.01, 0.05, and 0.1 are given.

*Risk at the NOAEL and the SNCD.* Distributions of the UER at the NOAEL, SNCD_1.0_, and SNCD_0.67_ are shown in Figure 2. Table 1 presents medians and lower 5th and upper 95th percentiles for the point estimate of extra risk, as well as the UER, at the NOAEL, SNCD_1.0_, and SNCD_0.67_. Based on the 786 selected data sets, the median of the UER at the NOAEL is 11%. The same median results when considering only the data set for which the NOAEL is lower than the highest dose (case 1b). Considering the 665 data sets for which an SNCD_0.67_ was derived, the median extra risk and UER at the NOAEL are 7% and 12%, respectively (case 1c; Table 1). Considering the 439 data sets for which the SNCD_1.0_ was derived, the median extra risk and UER at the NOAEL are higher: 9% and 14%, respectively (case 1d; Table 1). This indicates that the extra risk at the NOAEL is higher for data sets where an SNR ≥ 1 is observed within the experimental dose range (data sets with more pronounced dose–response relationships) compared with data sets where the signal is smaller than the noise (SNR < 1).

**Table 1:** Extra risk at the NOAEL and the SNCD, expressed as medians and lower 5th (P05) and upper 95th (P95) percentiles across the *n* data sets considered.

Point estimate of extra risk					UER**				
PoD	Case*a*	*n*	Median	P05	P95	Median	P05	P95
NOAEL		1		786		0.060		0.020		0.33		0.11		0.039		0.51
		1b		596		0.063		0.018		0.37		0.11		0.036		0.56
		1c		665		0.067		0.020		0.37		0.12		0.038		0.55
		1d		439		0.089		0.022		0.42		0.14		0.040		0.60
		2		133		0.061		0.022		0.43		0.11		0.038		0.63
		3		330		0.086		0.020		0.45		0.14		0.036		0.64
		4		456		0.055		0.022		0.17		0.096		0.040		0.28
SNCD_1.0_		1		439		0.11		0.026		0.44		0.18		0.038		0.63
		2		106		0.13		0.025		0.39		0.21		0.038		0.56
		3		217		0.19		0.028		0.50		0.30		0.040		0.73
		4		222		0.062		0.026		0.29		0.098		0.038		0.46
SNCD_0.67_		1		665		0.042		0.013		0.23		0.073		0.021		0.41
		2		124		0.045		0.013		0.25		0.083		0.021		0.41
		3		243		0.11		0.010		0.33		0.19		0.018		0.57
		4		422		0.029		0.013		0.12		0.047		0.022		0.23
**a**Different subsets of the 786 data sets were considered, as represented by the following cases. Case 1: All data sets adequate for dose–response modeling [according to the criteria in Supplemental Material, Section 1 (http://dx.doi.org/10.1289/ehp.1003327)] and for which an SNCD was derived; all 786 data sets adequate for modeling were included for the NOAEL case. Case 1b: Data sets in case 1 for which the NOAEL was lower than the highest dose. Case 1c: Data sets in case 1 for which an SNCD_0.67_ was derived. Case 1d: Data sets in case 1 for which an SNCD_1.0_ was derived. Case 2: Data sets with the lowest NOAEL in each selected technical report included in case 1. Case 3: Data sets in case 1 including at most one dose for which the observed extra risk was > 0 and ≤ 0.2; the observed extra risk at a given dose, *i*, was calculated as [*xi*/*ni* – *p*(0)]/[1 – *p*(0)], where *p*(0) is the point estimate of the background risk. Case 4: Data sets in case 1 including more than one dose for which the observed extra risk was > 0 and ≤ 0.2.																

The median of the UER at SNCD_1.0_ appears to be higher than that at the NOAEL (18% for SNCD_1.0_ case 1 vs. 14% for NOAEL case 1d; Table 1), whereas the median of the UER at SNCD_0.67_ appears to be lower than that at the NOAEL (7% for SNCD_0.67_ case 1 vs. 12% NOAEL case 1c). Thus, the SNCD_0.67_ and the SNCD_1.0_ bracket the NOAEL.

The UERs in Table 1 can be contrasted with standard BMRs (extra risk) of 1%, 5%, and 10%, typically employed in the BMD method. The UER at the NOAEL is > 1% (BMR > 0.01) in virtually all 786 data sets; it is > 5% for 90% of the data; and it is > 10% for 54% of the data (Figure 2). The corresponding values for the SNCDs are also given in Figure 2.

Table 1 also presents results for a subset of the selected data composed only of data sets with the lowest NOAEL across all end points considered in each selected technical report (case 2). This is intended to reflect the PoDs that would be likely considered in a practical risk assessment application. Only small differences are observed relative to results for the full data set (case 1). The extra risk associated with the NOAEL and the SNCDs becomes lower for data sets that are more informative regarding responses in the low-dose region (median risk for case 4 < median risk for case 3; Table 1); this effect is more pronounced for the SNCD than for the NOAEL.

**Figure 3:**
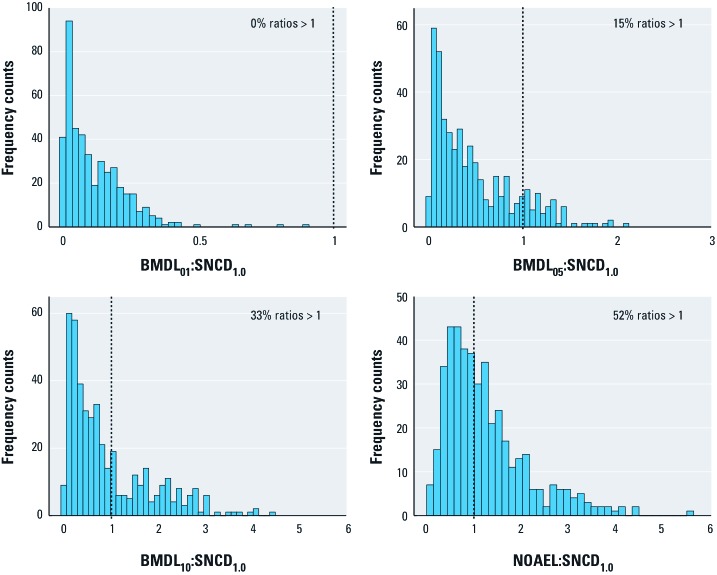
Ratios between different PoDs and the SNCD_1.0_. The dotted vertical lines indicate a ratio of 1.

**Figure 4:**
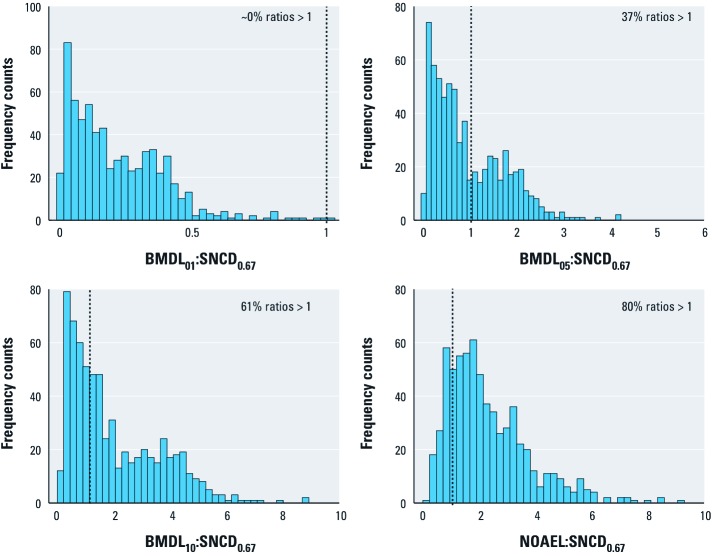
Ratios between different PoDs and the SNCD_0.67_. The dotted vertical lines indicate a ratio of 1.

*Comparison of the SNCD with the BMDL and NOAEL.* The distributions of BMDL:SNCD_1.0_ ratios are shown in Figure 3, along with the distribution of the NOAEL:SNCD_1.0_ ratios. Similar results are presented for the SNCD_0.67_ in Figure 4. The median and lower 5th and upper 95th percentiles of these distributions are summarized in Table 2. The BMDL_01_ is < SNCD_1.0_ for all data sets; the BMDL_05_ is > SNCD_1.0_ for 15% of the data sets; and the BMDL_10_ is > SNCD_1.0_ for 33% of the data sets (Figure 3). The median NOAEL:SNCD_1.0_ ratio is 1 (Table 2). The BMDL_01_ is < SNCD_0.67_ in virtually all data sets; the BMDL_05_ is > SNCD_0.67_ for 37% of the data sets; and the BMDL_10_ is > SNCD_0.67_ for 61% of the data sets (Figure 4). The median NOAEL:SNCD_0.67_ ratio is 1.9 (Table 2).

**Table 2:** Ratios between different PoDs and the SNCD, expressed as medians and lower 5th (P05) and upper 95th (P95) percentiles across the *n* data sets considered.

PoD:SNCD_1.0_							PoD:SNCD_0.67_						
PoD	Case*a*	*n*	Median	P05	P95	*n*	Median	P05	P95
BMDL_01_		1		439		0.082		0.0089		0.31		665		0.17		0.020		0.50
		2		106		0.062		0.0086		0.30		124		0.14		0.015		0.47
		3		217		0.043		0.0069		0.34		243		0.071		0.013		0.70
		4		222		0.14		0.020		0.27		422		0.23		0.048		0.48
BMDL_05_		1		439		0.34		0.046		1.3		665		0.71		0.11		2.3
		2		106		0.30		0.045		1.3		124		0.63		0.078		2.3
		3		217		0.19		0.036		1.2		243		0.31		0.069		1.8
		4		222		0.55		0.10		1.3		422		1.1		0.25		2.4
BMDL_10_		1		439		0.62		0.096		2.8		665		1.3		0.22		4.9
		2		106		0.58		0.095		2.8		124		1.2		0.17		4.9
		3		217		0.34		0.076		2.2		243		0.59		0.14		3.9
		4		222		1.0		0.22		2.8		422		2.1		0.49		5.1
NOAEL		1		439		1.0		0.31		3.1		665		1.9		0.56		5.2
		2		106		1.0		0.34		3.3		124		1.7		0.51		4.8
		3		217		1.0		0.28		3.2		243		1.6		0.42		5.0
		4		222		1.1		0.33		3.0		422		2.1		0.63		5.4
**a**Different subsets of the 786 data sets were considered, as represented by the following cases. Case 1: All data sets adequate for dose–response modeling [according to the criteria in Supplemental Material, Section 1 (http://dx.doi.org/10.1289/ehp.1003327)] and for which an SNCD was derived. Case 2: Data sets with the lowest NOAEL in each selected technical report included in case 1. Case 3: Data sets in case 1 including at most one dose for which the observed extra risk was > 0 and ≤ 0.2; the observed extra risk at a given dose, *i*, was calculated as [*xi*/*ni* – *p*(0)]/[1 – *p*(0)], where *p*(0) is the point estimate of the background risk. Case 4: Data sets in case 1 including more than one dose for which the observed extra risk was > 0 or ≤ 0.2.																		

Results for the subset of data composed of only data sets with the lowest NOAEL in each NTP technical report (case 2; Table 2) are similar to those presented above for all data sets (case 1). The SNCD tends to decrease, relative to the other PoDs, for data sets that are more informative regarding responses in the low-dose region (i.e., median ratios for case 4 > median ratios for case 3; Table 2); this effect is more pronounced in the BMDL:SNCD ratios than in the NOAEL:SNCD ratios.

**Figure 5:**
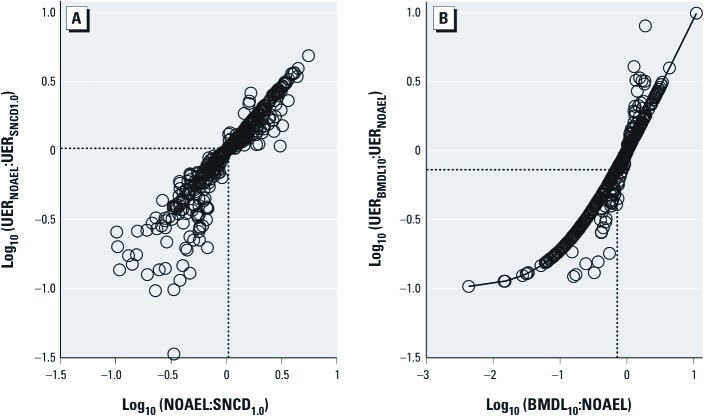
Correlations between differences in dose and UER. (*A*) Correlation between NOAEL:SNDC_1.0_ and UER_NOAEL_:UER_SNDC1.0_ based on the 439 data sets (circles) for which an SNCD_1.0_ was derived. The median dose and risk ratio, shown by the vertical and horizontal lines, are both 1. (*B*) Correlation between BMDL_10_:NOAEL and UER_BMDL10_:UER_NOAEL_, based on the 439 data sets (circles) for which an SNCD_1.0_ was derived. Observe that the UER at BMDL_10_ is 0.10. The median dose and risk ratio are 0.71 and 0.73, respectively (if all 786 data sets adequate for modeling are considered, the median dose and risk ratio are both 0.93). Circles in *B* situated along the curved line correspond to data sets for which the Hill coefficient η = 1; the other circles correspond to data sets for which η > 1.

*Correlations between differences in dose and risk.* The median UER at the SNCD_1.0_ is higher than that at the NOAEL (18% vs. 11–14%; Table 1), although the median NOAEL:SNCD_1.0_ ratio is 1 (Table 2), which may appear contradictory. The association between the ratio of the UERs at the NOAEL and SNCD_1.0_, and the ratio of the SNCD_1.0_ and NOAEL per se, is depicted in graphical form in Figure 5, which shows that the median of both ratios is 1. Thus, although the median UER at the SNCD_1.0_ and NOAEL differs (Table 1), the median ratio of the UERs is 1 (Figure 5A). This is due to differences in the shape of the respective UER distributions; the distribution is more skewed to the left for the NOAEL relative to the SNCD_1.0_ (Figure 2). The relationship between the BMDL_10_ and the NOAEL is portrayed in a similar fashion in Figure 5B. The circles in Figure 5B that are situated along the curved line correspond to data sets for which the Hill coefficient η = 1. Thus, the relation between the ratio of the UERs and the measurements themselves differs for data sets with η = 1 versus η > 1.

*Establishment of an RfD based on the NOAEL, BMDL, and SNCD.* The BMR_m_ at the NOAEL was previously determined to be slightly above 10% (11–14%, depending on data set selection; Table 1). This is the general level of risk associated with the traditional PoD in risk assessment, given the range of study designs covered in this analysis. Thus, the application of a standard UF of 100 to the BMDL_10_ can be expected to lead to RfDs that are, on average, similar to those based on the NOAEL.

**Figure 6:**
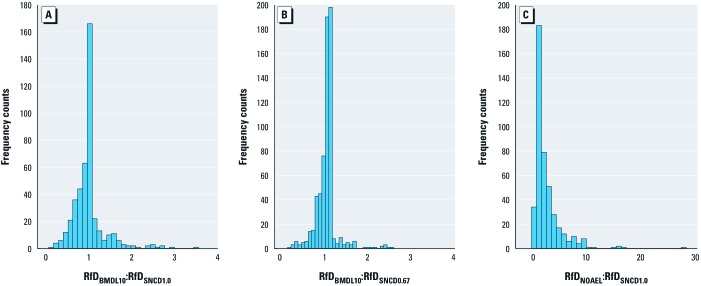
Comparison of different RfDs based on different PoDs. (*A*) The median RfD_BMDL10_:RfD_SNCD1.0_ ratio is 1; 50% of the ratios are > 1. (*B*) The median RfD_BMDL10_:RfD_SNCD0.67_ ratio is 1; 66% of the ratios are > 1. (*C*) The median RfD_NOAEL_:RfD_SNCD1.0_ ratio is 1.5; 72% of the ratios are > 1.

To compare RfDs based on the SNCD with RfDs based on the NOAEL and BMDL_10_, we assumed that the dose–response curve is linear below the PoD and that the RfD corresponds to a target risk in animals, which is 1/1,000 (i.e., BMR_m_/100, where BMR_m_ is set to 0.10). Under these assumptions, the RfD based on the SNCD was derived by linear extrapolation; that is, a linear model was drawn from the UER_SNCD_ down to zero extra risk, and the RfD was estimated as the dose corresponding to the target risk of 1/1,000 according to the linear model. Comparisons between RfDs based on the application of a UF of 100 to both the BMDL_10_ and the NOAEL with RfDs based on the SNCD, as described above, are tabulated in Table 3 and presented in graphical form in Figure 6. The medians of the ratios between RfDs based on the BMDL_10_ and the SNCD_1.0_ or SNCD_0.67_ both equal 1; the variability in the ratios across data sets is, however, generally less for the BMDL_10_:SNCD_0.67_ ratio than for BMDL_10_:SNCD_1.0_ (Table 3). The median of the ratio between RfDs based on the NOAEL and RfDs based on the SNCDs is 1.2 and 1.5 for SNCD_0.67_ and SNCD_1.0_, respectively (case 1); the variability in this ratio is much greater than for the BMDL_10_:SNCD ratios (Table 3).

**Table 3:** Ratios between RfDs based on the BMDL_10_ and NOAEL, and RfDs based on the SNCD, expressed as medians and lower 5th (P05) and upper 95th (P95) percentiles across the *n* data sets considered.

RfD ratio*a*	Case*b*	*n*	Median	P05	P95
RfD_BMDL10_: RfD_SNCD1.0_		1		439		1.0		0.50		1.6
		2		106		0.98		0.51		1.8
		3		217		0.89		0.39		1.7
		4		222		1.0		0.75		1.5
RfD_BMDL10_: RfD_SNCD0.67_		1		665		1.0		0.66		1.3
		2		124		1.0		0.66		1.1
		3		243		0.93		0.42		1.6
		4		422		1.1		0.84		1.1
RfD_NOAEL_: RfD_SNCD1.0_		1		439		1.5		0.41		7.6
		2		106		1.4		0.41		8.6
		3		217		2.4		0.65		9.3
		4		222		1.0		0.38		3.2
RfD_NOAEL_: RfD_SNCD0.67_		1		665		1.2		0.39		7.7
		2		124		1.2		0.40		9.9
		3		243		2.2		0.42		11
		4		422		1.0		0.39		3.3
**a**The RfDs based on the BMDL_10_ and NOAEL were established by dividing the respective PoD by a UF of 100; the RfD based on the SNCD was established by linear extrapolation from the UER_SNCD_,and it corresponds to a target extra risk of 1/1,000. **b**Different subsets of the 786 data sets were considered, as represented by the following cases: Case 1: All data sets adequate for dose–response modeling [according to the criteria in Supplemental Material, Section 1 (http://dx.doi.org/10.1289/ehp.1003327)], and for which an SNCD was derived. Case 2: Data sets with the lowest NOAEL in each selected technical report included in case 1. Case 3: Data sets in case 1 including at most one dose for which the observed extra risk was > 0 and ≤ 0.2; the observed extra risk at a given dose, *i*, was calculated as [*xi*/*ni* – *p*(0)]/[1 – *p*(0)], where *p*(0) is the point estimate of the background risk. Case 4: Data sets in case 1 including more than one dose for which the observed extra risk was > 0 and ≤ 0.2.										

Results for the subset of data composed of only data sets with the lowest NOAEL in each NTP technical report (case 2; Table 3) are similar to those presented above for all data sets (case 1). The BMDL_10_- versus SNCD-based RfD ratios tend to increase slightly for data sets that are more informative regarding responses in the low-dose region (case 4 vs. case 3), whereas the opposite holds for the NOAEL- versus SNCD-based RfD ratios (case 3 vs. case 4; Table 3). Considering the more informative data sets only (case 4), the median of all four ratios is approximately 1.

*Model dependence and impact of sample size.* To assess model dependence, results from the Hill model can be contrasted with those derived from the Weibull model [see Supplemental Material, Section 3 (http://dx.doi.org/10.1289/ehp.1003327)]. Model dependence is minimal for both the BMDL and the SNCD_0.67_ in both subsets of the data considered. Although this represents a somewhat limited analysis of model dependence, it does suggest that sensitivities of these two PoDs to the choice of model are similar. An increased sample size is beneficial for both the SNCD and the BMDL, so that larger experiments will lead to higher human exposure guidelines when using either of these PoDs; a greater benefit was observed for the SNCD-based exposure guideline relative to the BMDL-based exposure guideline (see Supplemental Material, Section 4).

## Discussion

In this article we address issues related to establishing human exposure guidelines for chemicals, comparing both existing (the NOAEL and the BMDL) and new (the SNCD) PoDs for determining an RfD. Empirical comparisons among RfDs based on these three PoDs were made using the complete NTP 2-year carcinogenesis bioassay database, including 786 quantal dose–response data sets suitable for dose–response modeling. The SNCD concept is an objective approach for determining the lowest dose that can be used as a PoD for determining a human exposure guideline, without having the signal overwhelmed by noise.

The selection of the most appropriate BMR to use in the application of the BMD approach has usually been addressed by comparing NOAEL and BMDL values over several data sets. The EFSA (2009a) pointed out in its scientific opinion on the BMD that for an individual NOAEL, the residual risk might be less than or greater than the BMR values (i.e., 1%, 5%, or 10%) normally considered in applications of the BMD concept; this point was illustrated by calculating the upper bound on the extra risk at the NOAEL for a limited selection of substances that have been previously evaluated. The present study provides a much more comprehensive picture of the possible level of residual at the NOAEL by calculating a UER_NOAEL_ across a large number of data sets (the UER_NOAEL_ estimated in this study translates to the BMR as it is used in the BMD approach). Specifically, we showed that the median of the UER_NOAEL_ was slightly higher than 10% (Table 1), whereas the UER_SNCD_ was 7% or 18%, depending on the stringency of the definition of the SNCD [i.e., whether the SNR is set equal to 1 (SNCD_1.0_) or 0.67 (SNCD_0.67_), the former being more stringent].

Some of the results presented here can be contrasted with those from earlier studies that have compared the BMD and NOAEL. For developmental toxicity data, BMDLs corresponding additional risks of 1%, 5%, and 10%, estimated using a Weibull model, were lower than the NOAEL, on average. These results were based on 407 data sets demonstrating significant trends in risk with increasing dose. The mean and median NOAEL:BMDL_10_ ratios were 2.9 and 2.0, respectively (Allen et al. 1994a); this finding indirectly suggests that the average risk at the NOAEL is > 10%. Risk assessment methods designed specifically for developmental toxicity testing have also been applied. Allen et al. (1994b) found that a BMDL corresponding to an additional risk of 5% resembled the NOAEL on average; based on 253 developmental toxicity data sets, the median NOAEL:BMDL_05_ ratio in that study was 0.96. Other studies have assessed the risk at the NOAEL in more direct terms. Gaylor (1992) calculated the observed risk at the NOAEL using the proportion of affected fetuses at the NOAEL, minus the proportion of affected fetuses in the control group, essentially a point estimate of additional risk using a model-free approach. That analysis was based on 236 developmental toxicity data sets reported in the scientific literature. The risk at the NOAEL for either dead/resorbed or malformed fetuses ranged from 0 to 4.5% and exceeded 1% in about 25% of the cases. In a theoretical analysis of how large the risk may be at the NOAEL, Leisenring and Ryan (1992) reported that the average extra risks at the NOAEL varied between 3% and 21%, depending on the experimental designs and shapes of Weibull dose–response curves considered. An important difference between the present analysis and those conducted by Gaylor (1992) and Leisenring and Ryan (1992) is that we considered an upper bound on the risk at the NOAEL, in addition to a point estimate of risk. For continuous data, analysis of 395 NTP data sets (rat and mouse data on body weight, liver and kidney weight, and red blood cell counts) indicated that the BMDL_05_ (defined as a 5% change in the mean response relative to background) was close to the NOAEL at median (Bokkers and Slob 2007; EFSA 2009a).

The SNCD as defined in this article was higher than other reference points that have been suggested for risk assessment (Table 2, Figures 3, 4). In particular, the BMDL_01_, but also the BMDL_05_, was generally lower than the SNCD. Depending on the stringency of the definition of the SNCD, the median BMDL_10_:SNCD ratio was either 0.6 or 1.3, for an SNR of 1 or 0.67, respectively. The primary motivation for proposing the use of the SNCD as a PoD for low-dose risk assessment is to start from the lowest possible point on the dose–response curve for which the signal in the data can be reliably detected. With both the BMDL_01_ and BMDL_05_ being below the SNCD, they may not be appropriate as default PoDs. The BMDL_01_ may be particularly problematic is this regard, being uniformly lower than the SNCD in virtually all cases considered; viewed another way, the upper bound of extra risk at the SNCD (UER_SNCD_) was also higher than 1% for virtually all the data (Figure 2). The median NOAEL:SNCD ratio was 1 when the SNCD was defined in terms of an SNR of 1.0 (Table 2, Figure 5).

For some of the 786 data sets, the SNCD_1.0_ (44%), SNCD_0.67_ (15%), BMDL_10_ (12%), and BMD_10_ (31%) were outside the experimental dose range. The relatively high proportion of data sets (12–44%) for which these quantities were not within the experimental dose range suggests that many data sets may in fact provide a quite limited basis for quantitative risk assessment; such situations can become more apparent when dose–response modeling approaches are applied compared with when a NOAEL approach is used.

The automated model selection approach used in the present study resulted in the use of a Hill coefficient η = 1 in most cases [see Supplemental Material, Section 1 (http://dx.doi.org/10.1289/ehp.1003327)]. This is a reflection of the information value in the data regarding the Hill coefficient and should not be seen as evidence that the dose response in the low-dose region is in fact linear; rather, it indicates only that data could be adequately described by models that assumed linearity. This type of model selection approach is embraced in the BMD method. In the automated modeling approach, we decided to use an objective method for determining the appropriate degree of parameterization because the three-parameter model was not suitable for all data sets. The width of the CI, for example, can change appreciably, depending on whether a two- or three-parameter model is used. More specifically, the CI may be tighter when a model with fewer parameters is used, and this can become relevant for the SNCD as well as the BMDL.

The standard UFs used in risk assessment may need to be modified when the PoD is defined on the basis of the BMD. For example, it has been discussed that an extra UF may be needed when using the BMD approach because this PoD, by design, corresponds to a nonzero risk equal to the BMR (U.S. EPA 1995). This argument is presented with the implicit assumption that the NOAEL is a true “no effect level.” However, because the median UER associated with the NOAEL was close to 10%, it could also be argued that the standard UFs might be as appropriate for the BMDL_10_ as they are for the NOAEL because the resultant RfDs would, on average, provide a similar level of protection.

If the dose–response curve is linear below the PoD, application of the default UF of 100 to the BMDL_10_ translates to a target (extra) risk of 1/1,000. Using this as a basis, we developed a modified RfD approach using the SNCD as the PoD: Specifically, the RfD is calculated as the dose corresponding to a target extra risk of 1/1,000 by linear extrapolation from the UER_SNCD_. Implicit in this approach is the application of a data-specific UF to the SNCD in order to arrive at the RfD. We used a target risk of 1/1,000 because it calibrates to applying the default UF of 100 to the more traditional PoDs (the NOAEL and BMDL_10_). This choice of target risk provided a starting point under which the different approaches could be compared. Further development of the SNCD concept could involve alternative target risks, based on public health considerations (for performing risk reduction using the SNCD as starting point), as well as alternative low-dose extrapolation models. Although not considered herein, animal-to-human extrapolation may also be regarded as a separate step after the SNCD-based PoD has been established. In the interim, the median RfD_BMDL10_:RfD_SNCD1.0_ ratio was approximately 1 (Table 3). The median RfD_NOAEL_:RfD_SNCD1.0_ ratio was > 1, except for the more informative data sets (Table 3).

Historically, the most common argument put forward in favor of the BMD as a PoD is the explicit recognition of sample size, which the NOAEL accounts for in an inappropriate manner. As the sample size increases, the uncertainty in the BMD will decrease, resulting in a less conservative RfD. This is clearly beneficial from a regulatory point of view because larger experiments then may allow higher (less conservative) exposure guidelines. However, the practical consequence of this theoretical advantage of the BMD approach, at the level of a resulting RfD, depends on how much the sample size is increased and possibly on the selected BMR. The SNCD will also be affected by an increasing sample size (theoretically, it will be decreased), and as shown in this article, it will also be affected by the “value of information” embedded in the data in the lower dose region (case 3 vs. case 4; Tables 1, 2). In case of a sublinear or linear dose–response below the SNCD, linear extrapolation from the SNCD will result in a less conservative RfD, as data quality increases. In the hypothetical case of a supralinear dose–response below the SNCD, however, the opposite applies. In any event, the extent to which low-dose extrapolation is necessary is reduced because the SNCD decreases as data quality increases. The impact of sample size on the SNCD is illustrated and discussed in detail in Supplemental Material, Section 4 (http://dx.doi.org/10.1289/ehp.1003327).

The empirical results presented in this article are based on cancer bioassay data from the NTP. Because these results focus on a specific toxicological end point assessed under protocols that follow a relatively standard experimental design, their generalizability to other end points is constrained. To address this limitation, and to further explore the SNCD concept, we plan to repeat the empirical analyses conducted in this study using other databases, beginning with the NTP database on high-throughput *in vitro* screens, which involves a wide range of biological end points reflecting different toxicity pathways. High-throughput screens are of particular interest in this regard because they represent one of the cornerstones of the U.S. EPA’s strategic plan for evaluating the toxicity of environmental agents in the future (Firestone et al. 2010; Krewski et al. 2011). Although we discuss the SNCD concept for quantal dose–response data in this article, the concept is also applicable to continuous dose–response data. Methodological extensions of the SNCD concept to accommodate continuous or polychotomous data will be pursued in future research.

## Conclusions

Using NTP 2-year carcinogenesis bioassay data as the basis for an empirical study of the properties of different PoDs for establishing human exposure guidelines to environmental agents, we found that the median risk associated with the traditional PoD for risk assessment—the NOAEL—is close to 10%. Given the type of study design covered in the analysis, this observation suggests that the application of standard UFs to the BMDL_10_ will provide a similar level of protection, at the median, as the application of those same factors to the NOAEL, when deriving an RfD. The SNCD proposed here also appears to warrant consideration as a reference point in risk assessment because it provides an objective estimate of the lowest dose that may reasonably serve as a PoD for low-dose risk assessment. Our analysis suggests that use of extra risks < 10% may not be appropriate as default BMRs because corresponding BMDLs were generally below the SNCD. For high-quality data sets (i.e., with larger sample size and/or more information in the low-dose range), the SNCD will decrease, which reduces the extent of extrapolation necessary. Using the default UF of 100 as a basis and noting that the median risk associated with the NOAEL is close to 10%, we suggest a target risk of 1/1,000 for derivation of an RfD from the SNCD by linear extrapolation. At the median, this approach provided the same RfD as the BMDL_10_ divided by the default UF of 100. Although a linear extrapolation model and a target risk of 1/1,000 were used for derivation of the SNCD-based human exposure guideline, other models for low-dose extrapolation, and target risks relevant from a public health point of view, may be considered as the SNCD concept is further developed.

## Supplemental Material

(262 KB) PDF
