# Supplementary material for: A Signal-to-Noise Crossover Dose as the Point of Departure for Health Risk Assessment
Source: Environ Health Perspect. 2011 Aug 3;119(12):1766–74. doi: 10.1289/ehp.1003327 (PMC3261975; doi:10.1289/ehp.1003327)
Supplement: (262 KB) PDF [file ehp.1003327.s001.pdf]

A Signal-to-Noise Crossover Dose as the Point of Departure for Health Risk Assessment

Supplemental Material

Salomon Sand*[1,2], Christopher J. Portier[3], Daniel Krewski[2,4]

[1] Risk Benefit Assessment Department, National Food Administration, Uppsala, Sweden

[2] McLaughlin Centre for Population Health Risk Assessment, University of Ottawa, Ottawa, Canada

[3] National Institute of Environmental Health Sciences; currently at National Center for Environmental Health and Agency for Toxic Substances and Disease Registry, Atlanta, GA, USA.

[4] Risk Sciences International, Ottawa, Canada

Table of Contents

1. Model fitting	3
2. Estimation of the signal-to-noise crossover dose (SNCD)	4
3. Analysis of model dependence	5
4. Influence of sample size on the SNCD and BMDL	6

1. Model fitting

The dose-response data was fitted with the Hill model:

$$p(d_i) = \alpha + (1-\alpha)\left(\frac{d_i^{\eta}}{\kappa^{\eta} + d_i^{\eta}}\right),$$

where α is a parameter that describes the background incidence; κ is the location parameter; η describes the shape of the dose-response curve; and d_i is the dose administered to the i'th treatment group. The background parameter was restricted to be, $\alpha > 0$, and the shape parameter was restricted to be, $\eta \geq 1$ and smaller than 10. For $\eta = 1$, the model is linear at low doses. Both two- and three-parameter versions of the Hill model were considered. The Hill model was fitted by maximum likelihood. The calculations were performed in Matlab (version 7.4). The specific steps involved in the analysis are described below:

1) The three-parameter Hill model was fitted to each dataset using the maximum likelihood approach. The two-parameter version of the model where, $\eta = 1$ was also fitted, and the preferred model was selected based on a likelihood ratio test at significance level, $p \leq 0.05$. The two-parameter model was directly preferred for datasets with 3 dose groups. For these datasets, the optimal integer value of η was derived: specifically, the two-parameter model was re-fitted with $\eta = 1, 2, 3, \ldots$ until it was not significantly different from the three-parameter model. This approach was also applied for some datasets where η approached very high values (which yields an unreasonable steep curve) due to lack of information in the data that constrains the shape of the fitted dose-response curve; datasets for which $\eta \geq 10$ were classified in this category.

2) To be included in the present analysis, the data needed to fulfill the standard requirements for BMD analysis. The Hill model selected in step (1) was compared to a horizontal line fitted to the data using a likelihood ratio test ($p \leq 0.05$) to determine if the data demonstrated a dose-response trend. To assess goodness-of-fit, the selected model was compared to the data (the saturated model) under a likelihood ratio test at significance level, $p \leq 0.05$. Datasets not satisfying these requirements were not used;

this resulted in the exclusion of an additional 342 datasets, leaving a total of 1128 - 342 = 786 datasets for analysis.

Among the 786 datasets, the two-parameter model with $\eta = 1$ was selected in 656 cases; the two-parameter model with $\eta > 1$ was selected in 68 cases (values of η in the range of 2 - 6, mostly 2, were obtained); and the three-parameter model was selected for the remaining 62 datasets.

The Weibull model was, in addition to the Hill model, applied in the analysis of model dependence (described in Supplemental Material, section 3):

$$p(d_i) = \alpha + (1-\alpha)\left(1 - e^{-\left(\frac{d_i}{\kappa}\right)^{\eta}}\right)$$

The parameters in the Weibull model can be interpreted similarly to those in the Hill model.

2. Estimation of the signal-to-noise crossover dose (SNCD)

The SNCD is defined as the dose where the point estimate of additional risk is equal to or, alternatively, 2/3 times the (absolute) difference between the upper and lower bound of a two-sided 90% confidence interval on absolute risk at that dose. The profile likelihood method was used for estimating the SNCD for each dataset using the algorithm described below.

1) Starting at the highest dose tested, the upper and lower bounds of a two-sided 90% confidence interval on absolute risk (P95 and P05) were determined using the profile likelihood method. The signal-to-noise ratio (SNR) for this dose, *d*, was then calculated as:

$$SNR(d) = \frac{p(d) - p(0)}{P95 - P05},$$

where $p(d) - p(0)$ is the point estimate of the additional risk.

2) Step (1) was repeated sequentially for a lower dose (the dose, *d*, was lowered by a small factor in each round) until the SNR was smaller than the specified critical value. Critical values of 1 and 2/3 were considered.

3) The relationship between dose and the SNR was modeled by spline interpolation between the data points generated in steps (1) and (2). The SNCD was then calculated as the dose where the SNR equals the critical value of 1 or 0.67: these doses were labeled $SNCD_{1.0}$ and $SNCD_{0.67}$, respectively. The additional risks at these doses were also calculated; i.e. the signal-to-noise crossover responses, $SNCR_{1.0}$ and $SNCR_{0.67}$, respectively.

Application of dose-response modeling approaches in risk assessment requires availability and user friendliness of appropriate software. To be more generally applicable, the SNCD would need to be implemented as a choice in available software. From a practical point of view, the extension of estimating the SNCD relative to the BMDL does not imply much longer execution time (but, if several 100 datasets are analyzed, like in this study, it will take a somewhat longer time to calculate the SNCD compared to the BMDL).

3. Analysis of model dependence

Results from the Hill model were contrasted to those derived from the Weibull model. This analysis was performed for two subsets of the data, specifically: (1) "Case 2: $SNCD_{0.67}$", comprising 124 datasets for which the $SNCD_{0.67}$ was derived (124 datasets with the lowest NOAEL in each selected NTP technical report; for most of these datasets the two-parameter models was selected), and (2) "Case 1: 3p model", comprising all datasets for which the three-parameter Hill model was selected (62 datasets; see Supplemental Material, section 1).

For a given PoD (SNCD or BMDL), model dependence (MD) was defined as the ratio between highest and lowest estimate derived from the Hill and Weibull models. Model dependence was minimal for both subsets of data. For the 124 "Case 2: $SNCD_{0.67}$" datasets, MD was higher than a factor 2 for only 7 ($SNCD_{0.67}$), 9 ($BMDL_{05}$), and 8 ($BMDL_{10}$) datasets, depending on the PoD under consideration. For the 62 "Case 1: 3p model" datasets, MD was always lower than a factor 2. As shown in Supplemental Material, Table 1, the degree of MD in the BMDL is similar to that of the $SNCD_{0.67}$; the MD-ratio appears to be quite evenly

distributed around the value of 1 (for an MD-ratio = 1, MD is the same for both PoDs). While this represents a somewhat limited analysis of MD, it does suggest that the two types of PoDs are not highly dependent to the choice of the dose-response model used to describe the experimental data, and that sensitivities of these PoDs to the choice of model are similar.

Supplemental Material, Table 1. Difference in model dependence (MD) between the BMDL and the $SNCD_{0.67}$.

MD-ratio	Case	N	Median	P05	P95	N for which MD-ratio > 1
$MD_{BMDL05}/MD_{SNCD0.67}$	Case 2: $SNCD_{0.67}$	124	1.0	0.57	1.5	100
$MD_{BMDL10}/MD_{SNCD0.67}$	Case 2: $SNCD_{0.67}$	124	1.0	0.53	1.4	71
$MD_{BMDL10}/MD_{SNCD0.67}$	Case 1: 3p model	62	1.0	0.79	1.2	31

Note: Model dependence (MD) was defined as MD = $PoD_{highest}/PoD_{lowest}$, where PoD is the SNCD or BMDL estimated by either the Hill or Weibull models. Medians, lower 5th (P05) and upper 95th (P95) percentiles are presented for the MD-ratio. The number of datasets, N, for which the MD-ratio > 1 represents the number of datasets where MD is smaller for the SNCD than for the BMDL.

4. Influence of sample size on the SNCD and BMDL

This section illustrates the dependency of the SNCD and the SNCD-based exposure guideline on sample size. Dose-response data on the incidence of hepatocellular carcinoma observed in male F344/N rats exposed to furan (NTP Technical Report No. 402) is used in this example. The observed data with n = 50 animals per group, as well as datasets constructed by extrapolating the observed incidence rates in the original experiment to different sample sizes, n, are shown in Supplemental Material, Table 2. In particular, four theoretical datasets with n = 10, 25, 75, and 100 animals per group were constructed by multiplying the observed incidence rates by a common factor of 0.2, 0.5, 1.5, or 2. In the event that the numerator in the adjusted incidence (i.e., the number of animals with hepatocellular carcinoma) was not a whole number, the numerator was rounded to the nearest lower integer. As a consequence, the fraction of responding animals at 2, 4, and 8 mg/kg in theoretical dataset 1 is somewhat

different compared to that for the observed data, and the fractions of responding animals at 2 mg/kg in theoretical datasets 2 and 3 are somewhat different to that in the observed data. The observed and theoretical datasets were modeled with the three-parameter Hill model using the approaches described in Supplemental Material, sections 1 and 2.

Supplemental Material, Table 2. Observed and theoretical dose-response data on the incidence of hepatocellular carcinoma observed in male F344/N rats exposed to furan (NTP Technical Report No. 402).

Dose (mg/kg)	Theoretical dataset 1 (n=10)	Theoretical dataset 2 (n=25)	Observed dataset (n=50)	Theoretical dataset 3 (n=75)	Theoretical dataset 4 (n=100)
0	0/10	0/25	0/50	0/75	0/100
2	0/10	0/25	1/50	1/75	2/100
4	1/10	3/25	6/50	9/75	12/100
8	3/10	9/25	18/50	27/75	36/100

Note: Theoretical data has been constructed by extrapolating observed incidences to different sample sizes, n (the number of animals per group).

The $SNCD_{0.67}$ is shown in Supplemental Material, Figure 1, for the observed data and theoretical dataset 1; as the sample size is decreased from 50 to 10 animals per group, the SNCD increases by a factor 2.25.

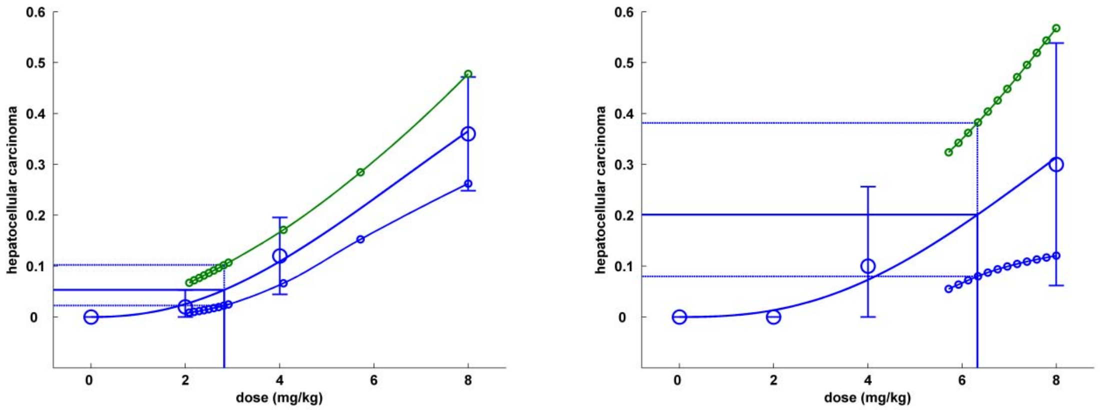

Supplemental Material, Figure 1. The three-parameter Hill model fitted to dose-response data on the incidence of hepatocellular carcinoma observed in male F344/N rats exposed to furan (NTP Technical Report No. 402). The left panel shows the $SNCD_{0.67}$ (2.8 mg/kg) for the observed data, and the right panel shows the $SNCD_{0.67}$ (6.3 mg/kg) for a theoretical dataset constructed by adjusting observed incidences to reflect a sample size of 10 animals per group (Supplemental Material, Table 2, theoretical dataset 1).

The relationship between sample size and the $SNCD_{0.67}$ and $BMDL_{10}$, respectively, is illustrated in Supplemental Material, Figure 2 (left part). As sample size increases, the SNCD decreases, while the opposite applies for the BMDL. The effect of sample size over the range considered (n = 10, 25, 50, 75 or 100) is higher for the SNCD than for the BMDL. In Supplemental Material, Figure 2 (right panel) the impact of sample size on the human exposure guidelines derived from the $SNCD_{0.67}$ and $BMDL_{10}$ is also illustrated. The SNCD-based guideline is the dose corresponding to a 1/1000 target risk, for the experimental animal, based on linear extrapolation from the upper bound on extra risk at $SNCD_{0.67}$. The $BMDL_{10}$-based exposure guideline corresponds to the application of a default uncertainty factor of 100 to the $BMDL_{10}$ (also corresponding to a 1/1000 target risk, for the experimental animal, according to linear extrapolation from the $BMDL_{10}$).

An increased sample size, n, leads to higher exposure guidelines for both the SNCD and $BMDL_{10}$ (Supplemental Material, Figure 2). The effect of sample size is more pronounced for the SNCD-based exposure guideline than for the BMDL-based exposure guideline. For both exposure guidelines, a change in sample size from 10 to 50 animals per group has a similar effect: the exposure guideline is increased by a factor 1.5-1.6 (a change which may not be

regarded as large in practice). A change in sample size from 50 to 100 animals per group has a greater effect for the SNCD-based exposure guideline compared to the BMDL-based exposure guideline, and depends on the dataset considered.

The effect of increasing the number of animals per group from 50 to 100 was further investigated in a subset of the data analyzed, comprised of all 62 datasets for which the three-parameter model was selected. The impact of this increase in sample size was higher for the SNCD-based exposure guideline relative to the BMDL-based exposure guideline. At median, the SNCD-based exposure guideline increased by a factor 1.4 (90% confidence interval: 1.2 - 1.8) due to this increase in sample size, with the corresponding increase for the BMDL-based exposure guideline being 1.1 (90% confidence interval: 1.06 - 1.3).

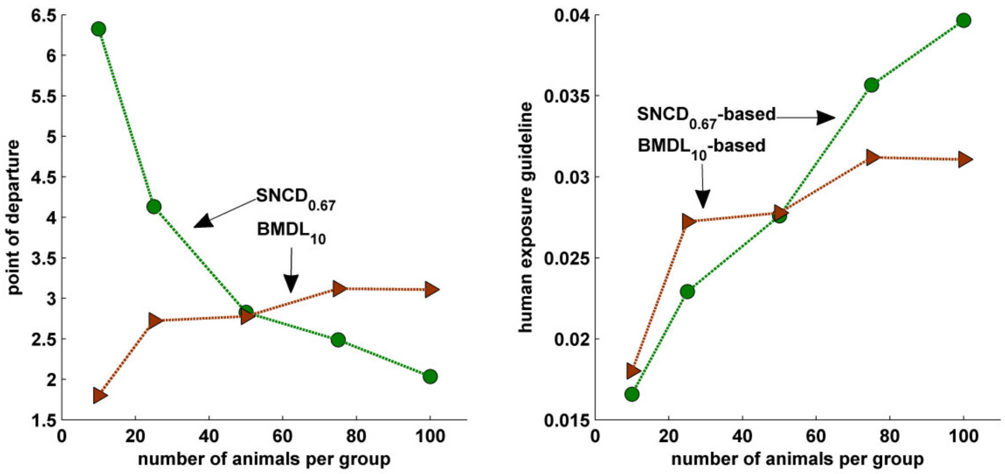

Supplemental Material, Figure 2. The left panel shows the relationship between sample size and the $SNCD_{0.67}$ and the $BMDL_{10}$; the right panel shows the same relationships at the level of established human exposure guidelines. The SNCD-based guideline is the dose corresponding to a 1/1000 target risk (for the experimental animal) according to linear extrapolation from the upper 95% confidence bound on extra risk at $SNCD_{0.67}$. The $BMDL_{10}$-based exposure guideline corresponds to the application of a default uncertainty factor of 100 to the $BMDL_{10}$. Values of the SNCD, the BMDL, and the human exposure guidelines are in mg/kg (administered 5 days/week, according to feeding schedule).
